# Carbon Dots: A Versatile Platform for Cu^2+^ Detection, Anti-Counterfeiting, and Bioimaging

**DOI:** 10.3390/molecules29174211

**Published:** 2024-09-05

**Authors:** Qian Wang, Xinyi He, Jian Mao, Junxia Wang, Liangliang Wang, Zhongchi Zhang, Yongfei Li, Fenglin Huang, Bin Zhao, Gang Chen, Hua He

**Affiliations:** 1 Shaanxi University Engineering Research Center of Oil and Gas Field Chemistry, Xi’an Shiyou University, Xi’an 710065, China18091276351@163.com (Z.Z.);; 2 Shaanxi Province Key Laboratory of Environmental Pollution Control and Reservoir Protection Technology of Oilfields, Xi’an Shiyou University, Xi’an 710065, China; 3 Shaanxi Engineering Research Center of Green Low-Carbon Energy Materials and Processes, Xi’an Shiyou University, Xi’an 710065, China; 4 State Key Laboratory of Heavy Oil Processing, Center for Bioengineering and Biotechnology, China University of Petroleum (East China), Qingdao 266580, China; 5 PetroChina Changqing Petrochemical Company, Xi’an 710032, China; 6 Department of Statistics, North Dakota State University, Fargo, North Dakota, ND 58102, USA

**Keywords:** carbon dots, Cu^2+^ detection, anti-counterfeiting, cell imaging

## Abstract

Carbon dots (CDs) have garnered extensive interest in basic physical chemistry as well as in biomedical applications due to their low cost, good biocompatibility, and great aqueous solubility. However, the synthesis of multi-functional carbon dots has always been a challenge for researchers. Here, we synthesized novel CDs with a high quantum yield of 28.2% through the straightforward hydrothermal method using Diaminomaleonitrile and Boc-D-2, 3-diaminopropionic acid. The size, chemical functional group, and photophysical properties of the CDs were characterized by TEM, FTIR, XPS, UV, and fluorescence. It was demonstrated in this study that the prepared CDs have a high quantum yield, excellent photostability, and low cytotoxicity. Regarding the highly water-soluble property of CDs, they were proven to possess selective and sensitive behavior against Cu^2+^ ions (linear range = 0–9 μM and limit of detection = 1.34 μM). Moreover, the CDs were utilized in fluorescent ink in anti-counterfeiting measures. Because of their low cytotoxicity and good biocompatibility, the CDs were also successfully utilized in cell imaging. Therefore, the as-prepared CDs have great potential in fluorescence sensing, anti-counterfeiting, and bioimaging.

## 1. Introduction

Fluorescent materials have emerged as a cutting-edge and rapidly advancing field in the past few years, revolutionizing analytical techniques and ushering in a new era of innovation [1]. In particular, when compared with some traditional techniques, current fluorescence analysis techniques have been significantly improved for the advantageous use of newly developed fluorescent materials. For example, researchers have developed various fluorescent materials, including fluorescent organic materials [2], oligomeric fluorescent materials [3], metal–organic-framework-based fluorescent materials [4], semiconductor quantum dots, and carbon dots (CDs) [5]. And they have applied them in different fields, such as metal ion detection [4,6,7], photocatalysis [8,9], drug delivery [10,11], information encryption [12,13,14], and bioimaging [15,16,17,18]. Apart from CDs, most of these fluorescent materials are complicated to prepare, and tedious fabrication steps are needed to functionalize them. Moreover, some of them are highly toxic, have poor stability, are easily photobleached, and have low fluorescence. Thus, it is highly necessary to develop fluorescent materials with a simple preparation method and explore their superior performance.

CDs have emerged as a fascinating type of fluorescent nanomaterial with sizes ranging from a few nanometers to several nanometers [19]. Due to their unique and excellent properties of low toxicity, simple surface functionalization, abundant raw material sources, low cost, tiny particle size, good fluorescence stability, tunable emission wavelength, and biocompatibility, diverse applications have been found for CDs across a wide range of fields, encompassing solar cells [20,21], bioimaging [22,23], chemo- and biosensors [24,25], anti-counterfeiting [26,27], and catalysis [28]. And numerous methods have been proposed to synthesize CDs, namely top-down and bottom-up methods [29,30]. The former refers to the cutting or peeling of larger carbon materials, such as carbon powder, graphite rods, carbon nanotubes, graphene, etc., to obtain CDs with the largest nanometer volume [31]. The latter is a method of aggregating a large number of tiny organic carbon atoms to form carbon nanoparticles [32,33]. Generally, organic or small-molecule oligomers are selected as the main carbon source. By using these methods, the size, morphology, structure, fluorescence, and surface chemistry of desired CDs can be controlled. Among these, the hydrothermal method is the most common, environmentally friendly, low-cost, and simple method for synthesizing CDs with the bottom-up approach [34]. This method usually uses organic acids, organic small molecules, polysaccharides, and some waste fruit peels as carbon sources and can be applied to prepare CDs with a high quantum yield (QY) [1,19].

The large quantity of recent studies on this topic has revealed that much effort has been made to fabricate CDs with superior performance for their application in various fields. For instance, Liu et al. [35] prepared nitrogen-doped CDs through the hydrothermal carbonization of citric acid and p-phenylenediamine, which demonstrated selective and sensitive properties for nitrite and a high efficiency for cell imaging with multicolor fluorescence. Xian et al. [36] synthesized red CDs with narrow dual emissions, a high fluorescence QY, and a high yield through the hydrothermal approach, using o-phenylenediamine and inorganic oxidants. They revealed the logarithmic relationship between the aggregation-induced emission wavelength and the concentration. Li et al. [37] used carboxymethyl nanocellulose as a carbon source and fabricated four types of CDs doped with different elements. Among them, N-doped CDs showed great sensitivity and selectivity for Hg^2+^ detection and exhibited great potential for anti-counterfeiting and information encryption and decryption. Li et al. [38] proposed a rapid microwave-assisted green synthesis method to prepare guanine-derived CDs and selectively detected the Ag^+^ in an aqueous solution. Wu et al. [39] simultaneously reported on 5.6 nm dual-emission CDs (DCDs) and 7.7 nm single-emission CDs (SCDs). The DCDs emitted at 489 and 676 nm when excited at 420 nm and were applied for the ratiometric determination of Cu^2+^. The SCDs emitted at 440 when excited at 365 nm and performed well in the determination of tetracyclines. Additionally, their great photostability, low toxicity, and excellent biocompatibility made these two types of CDs appropriate for multicolor cell imaging. Seesuea et al. [40] used water hyacinth as a starting material, which contains cellulose, hemicellulose, and lignin, and innovated a green CD synthesis approach by way of simple gamma irradiation. Then, they functionalized the surface functional groups of CDs with the thiol moiety, through which the CDs could serve as the fluorescent sensor to detect Hg^2+^, Fe^3+^, and Cu^2+^ ions at the micromolar level. Gedda et al. [41] synthesized CDs with prawn shells and applied them to Cu^2+^ detection in an environmental water sample. Thus, lowering the detection limits of metal ions is an important area for further research and improvement. All these works express that the precursors and the synthesis route can affect the chemical and physical properties of CDs, as well as their fluorescence and application. In addition, few CDs have been applied simultaneously in metal detection, anti-counterfeiting fluorescence, and cell imaging. Thus, developing multifunctional CDs and expanding their application in diverse fields is a common goal for researchers.

In this work, we have prepared a novel type of CD exhibiting multi-color fluorescence by a facile hydrothermal method, utilizing Diaminomaleonitrile and Boc-D-2, 3-diaminopropionic acid. The size, chemical groups, and photophysical properties of CDs were characterized using Transmission Electron Microscopy (TEM), Fourier Transform Infrared Spectroscopy (FTIR), X-ray Photoelectron Spectroscopy (XPS), Ultraviolet spectroscopy (UV-Vis) and fluorescence spectroscopy. It has been demonstrated that the as-prepared CDs have a high quantum yield, excellent photostability, and low cytotoxicity. Due to the highly water-soluble property of CDs, it has been proven that they possess selective and sensitive behavior towards Cu^2+^ ions. Moreover, CDs have been utilized as the fluorescence ink and the information encryption and anti-counterfeiting applications were easily achieved. Because of their low cytotoxicity and good biocompatibility, CDs have also been successfully utilized in cell imaging.

## 2. Results and Discussion

### 2.1. The Preparation and Characterization of CDs

**Figure 1:**
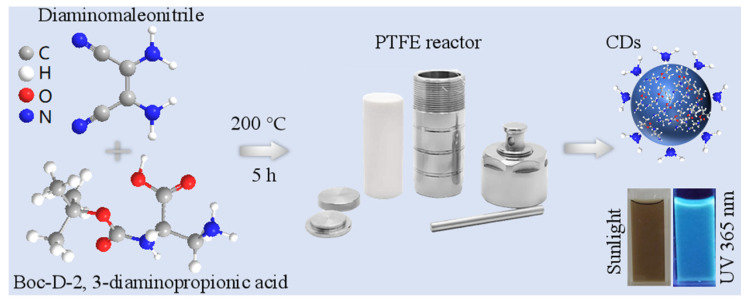
The schematic diagram of the synthesis process of the CDs.

The novel CDs were synthesized using the hydrothermal method, which is a simple and low-cost strategy. Boc-D-2, 3-diaminopropionic acid (DPA) and Diaminomaleonitrile (DAMN) were used as precursors in a ratio of 1:1 in water. Upon heating to 200 °C and keeping for 5 h in a sealed vessel, condensation and carbonization processes occurred to prepare CDs, which exhibit great dispersibility and stability in water at room temperature (Figure 1). The color of the CD solution under sunlight was light brown. However, a bright blue color fluorescence was emitted from the CD solution under the illumination of 365 nm UV light, which could be observed with the naked eye.

**Figure 2:**
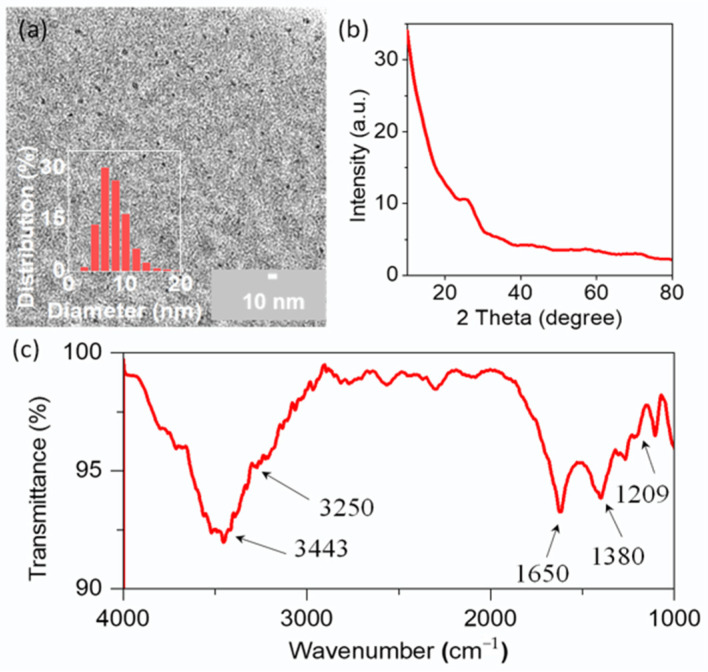
(**a**) TEM image and particle size distribution measured from TEM images of CDs; (**b**) XRD profile of CDs; (**c**) FT-IR spectra of CDs.

The morphology and size of the CDs were characterized by TEM, as shown in Figure 2a. They were nearly spherical carbon nanoparticles, approximately 7.5 nm in diameter with a narrow size distribution. The XRD profile of the CDs exhibited a peak at 25°, which corresponded to the crystal lattice distance of (002); this is a key feature of materials based on carbon (Figure 2b) [42,43]. Fourier transform infrared (FT-IR) analysis was carried out to probe the surface functional groups on a CD particle (Figure 2c). The peaks located at around 3443 and 3250 cm^−1^ could belong to the stretching frequencies of O–H and N–H. The sharp peak at 1650 cm^−1^ corresponded to the stretching vibration peak of the C=O bond, while those peaks around 1380 and 1209 cm^−1^ were attributed to C–N and C–O, respectively. These results verify that both the amine and carboxy groups exist on the particle surface of CDs as functional groups, which provides a possibility for further application research on CDs. The zeta potential of the CDs was estimated to be −6.5 mV, revealing that negatively charged groups are the majority.

**Figure 3:**
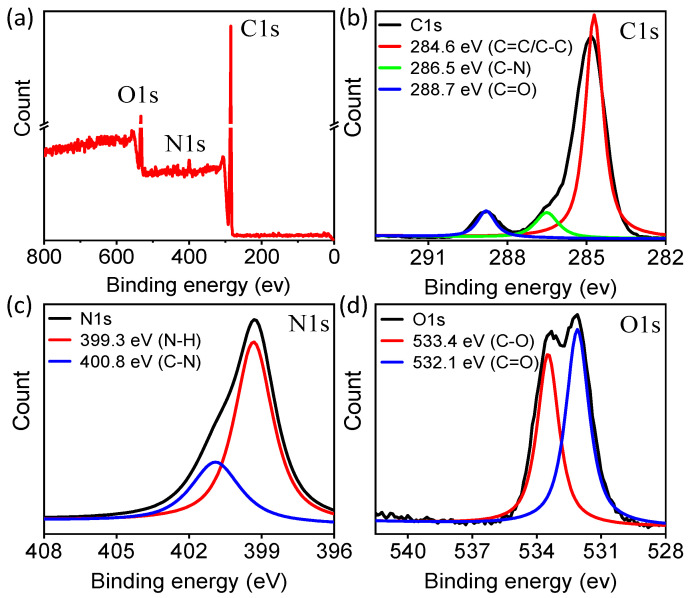
XPS spectra of DBP-CDs. (**a**) Survey profile, (**b**) C1s, (**c**) N1s, and (**d**) O1s.

To further certify the chemical structure of the synthetic CDs, X-ray photoelectron spectroscopy (XPS) analysis was also performed. The survey spectrum (Figure 3a) exhibits three strong peaks at 285 eV, 400 eV, and 532 eV, which can be attributed to C1s, N1s, and O1s. Each atomic percentage was 81.88%, 2.41%, and 15.71%, respectively, which indicates that the CDs were mainly composed of C, N, and O atoms. Deconvolution of the C1s spectrum (Figure 3b) reveals three peaks at 284.6, 286.5, and 288.7 eV, which correspond to C–C (C=C), N–C=N, and C=O groups, respectively. The N1s spectrum (Figure 3c) can be resolved into two components at 399.3, and 400.8 eV, associated with N–C=N and N–O bonds, respectively, verifying the presence of nitrogen in the carbon ring. The results indicate that the reactant took part in the reaction. For O1s, the deconvolution of the binding energy yielded two peaks, C=O (532.1 eV) and C–O (533.4 eV), as shown in Figure 3d. This XPS result is also consistent with the FTIR spectrum, suggesting that the amine exists in our CDs.

### 2.2. Optical Performance of the CDs

**Figure 4:**
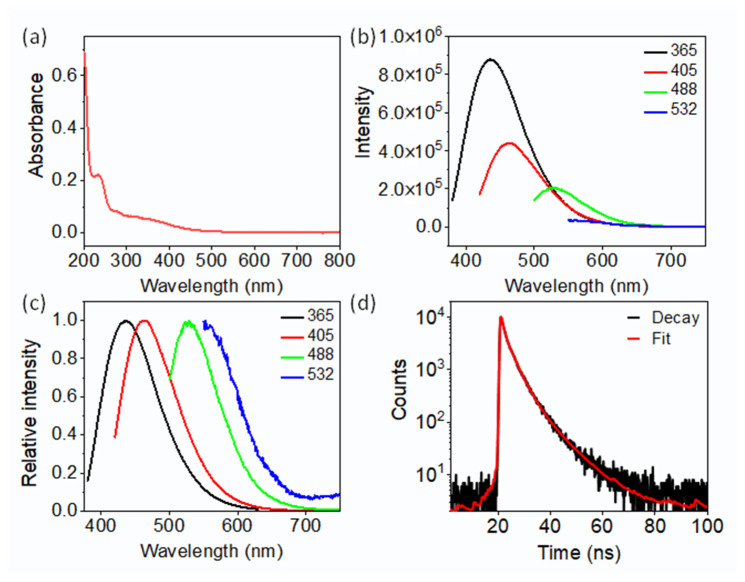
(**a**) UV–vis absorption and (**b**) fluorescence spectra of CD solution; (**c**) normalized fluorescence spectra of CDs solution. (**d**) Photostability of CDs under 532 nm laser, fluorescence decay curves (black), and fitted curves (red) of CDs.

UV–visible absorption spectroscopy was performed to investigate the photophysical properties of CDs (Figure 4a). The UV–vis spectrum of CDs exhibited wide optical absorption, ranging from 200 to 700 nm, with two exciton peaks observed at approximately 237 nm and 350 nm. The first peak at 237 nm indicates π–π* transition of C=C, and the second exciton peak around 350 nm is attributable to an n-π* transition of C=O. This suggests the condensation of the molecular precursors and the formation of CDs. To explore the fluorescence properties of CDs in detail, different excitation wavelengths were investigated (Figure 4b,c). The highest emission at 439 nm was observed using an excitation wavelength of 365 nm. Further, the quantum yield (QY) of the CDs was computed to be 28.2%, using quinine sulfate as a reference (ex = 370 nm, QY = 54%) [1]. The maximum emissions of 463 nm, 527 nm, and 554 nm were observed at excitation wavelengths of 405 nm, 488 nm, and 532 nm, indicating a distinct excitation-dependent feature of CDs. As previously reported, the blue fluorescence excited by UV light arises from molecular fluorophores, while the excitation-dependent fluorescence at the excitation wavelengths of >400 nm is attributable to surface states. Although the absorbance from surface states is relatively weak in the visible spectrum, they are beneficial for bioimaging due to the emission of long wavelength fluorescence. The fluorescence lifetime measurement of the CDs presents a multi-exponential fluorescence decay curve with an average lifetime of 3.7 ns.

**Figure 5:**
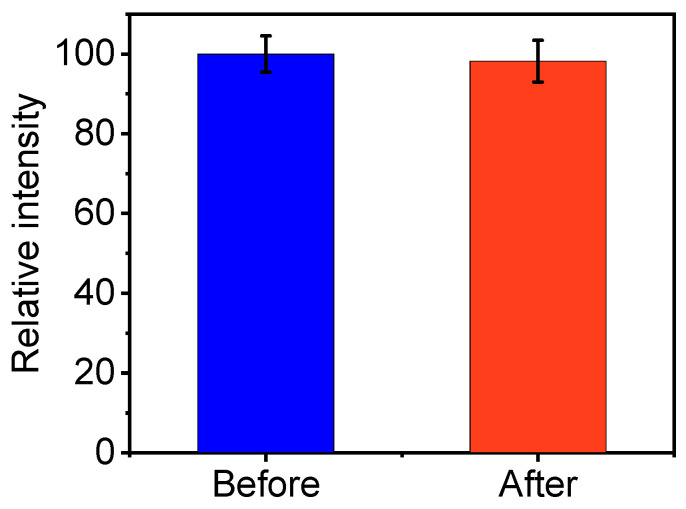
The fluorescence intensity of CDs before and after UV lamp irradiation at 365 nm for 40 min.

One of the key components in determining the optical characteristics of fluorescent probes is photostability. Therefore, the fluorescence fluctuation of the CDs was observed both before and after they were exposed to a 365 nm UV lamp. As shown in Figure 5, almost no obvious fluctuation in fluorescence intensity was observed from non-irradiation to irradiation within 40 min. This result implies the excellent photostability of the synthetic CDs.

### 2.3. Detection of Cu^2+^

**Figure 6:**
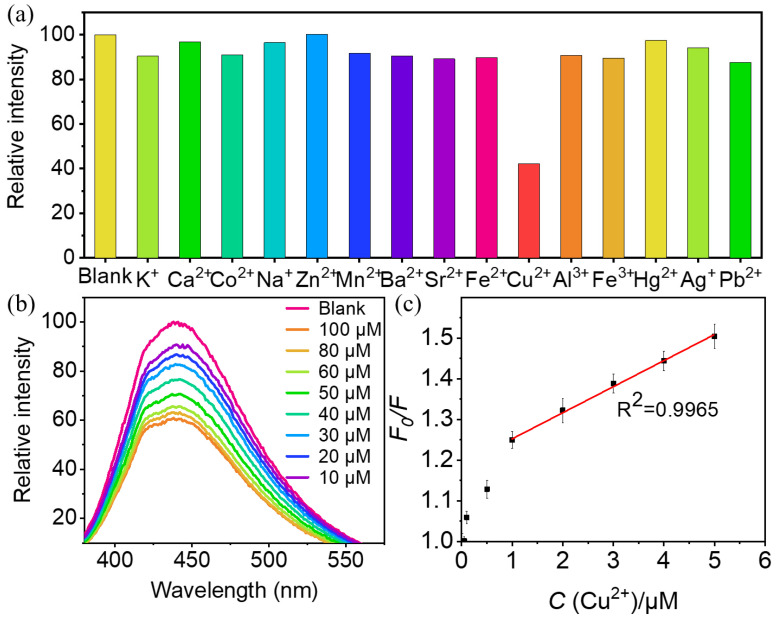
(**a**) Fluorescence responses of CDs in the presence of different metal ions; (**b**) fluorescence emission spectra of CDs with different concentrations of Cu^2+^ (from 10 µM to 100 µM); (**c**) linear relationship between *F*_0_/*F* and Cu^2+^ concentration within the concentration range of 0–9 µM.

Fluorescence sensing based on the fluorescent CD probe has attracted widespread attention for the detection of metal ions. Thus, we assessed the synthetic CDs as a sensor for different metal ions, including K^+^, Ag^+^, Ca^2+^, Co^2+^, Na^+^, Zn^2+^, Hg^2+^, Pb^2+^, Mn^2+^, Ba^2+^, Sr^2+^, Fe^2+^, Cu^2+^, Al^3+^, and Fe^3+^. Figure 6a displays the change in the fluorescence intensity of CDs after the addition of different metal ions. Surprisingly, the addition of copper ions resulted in a sharp decline in the intensity of fluorescent CDs, while other metal ions exhibited an inappreciable effect on fluorescence intensity. This phenomenon strongly confirms that synthetic CDs can selectively identify Cu^2+^ and can serve as a “turn-off” fluorescent probe for monitoring Cu^2+^. In order to eliminate the interference of other cations, we carried out an interfering experiment and investigated the most common interfering cations, including K^+^, Ag^+^, Ca^2+^, Co^2+^, Na^+^, Zn^2+^, Hg^2+^, Pb^2+^, Mn^2+^, Ba^2+^, Sr^2+^, Fe^2+^, Al^3+^, and Fe^3+^. This has validated that the addition of interfering cations did not induce any significant influence on the detection of Cu^2+^. Furthermore, to determine the impact of concentrations on fluorescence intensity, Cu^2+^ ions ranging from 10 μM to 100 μM were added to the CDs and the fluorescence was recorded (Figure 6b). With the increase in the concentration of copper ions, the fluorescence intensity of CDs reduced gradually. To examine the sensitivity of CDs to the concentrations of Cu^2+^, the fluorescence quenching efficiency was probed through the Stern–Volmer of *F*_0_*/F* = 1 + *K_SV_* [*C*] plot (R^2^ = 0.9965) in the concentration range of 0.1–9 μM (Figure 6c), where *F*_0_ and *F* are the intensities of fluorescent CDs before and after the addition of Cu^2+^ ions, [*C*] depicts the concentration of Cu^2+^, and the quenching constant *K_SV_* is calculated to be 0.063, which is the slope of the fitting line. Meanwhile, the limit of detection (LOD) of Cu^2+^ was measured to be 1.38 μM based on the formula mentioned in Section 3.7. This result is much lower than those of previous reports listed in Table 1, indicating that the proposed Cu^2+^ probe is more sensitive. Compared with previous reports, this presented method is simple, sensitive, selective, and enables the label-free sensing of Cu^2+^. Synthetic CDs can be implemented as a highly sensitive fluorescent sensor to quantitatively monitor Cu^2+^. In terms of the Cu^2+^ detection mechanism of CDs, firstly, Cu^2+^ exhibits the highest absorption affinity to the carbon nanostructure compared to other transition metals. This is the prerequisite for the effective fluorescence quenching in the CDs/Cu^2+^ system [44]. Secondly, it should be attributed to the electron transfer from excited CDs to the unfilled orbital of Cu^2+^, which further leads to strong non-radiative electron/hole recombination, resulting in aggregation-induced emission quenching (AIE) in CDs-Cu^2+^ [45]. Thirdly, due to the excellent oxidizing properties of Cu^2+^, this makes copper ions the most adequate receptors for the transferred electron from CDs [46].

### 2.4. CDs for Anti-Counterfeiting

**Figure 7:**
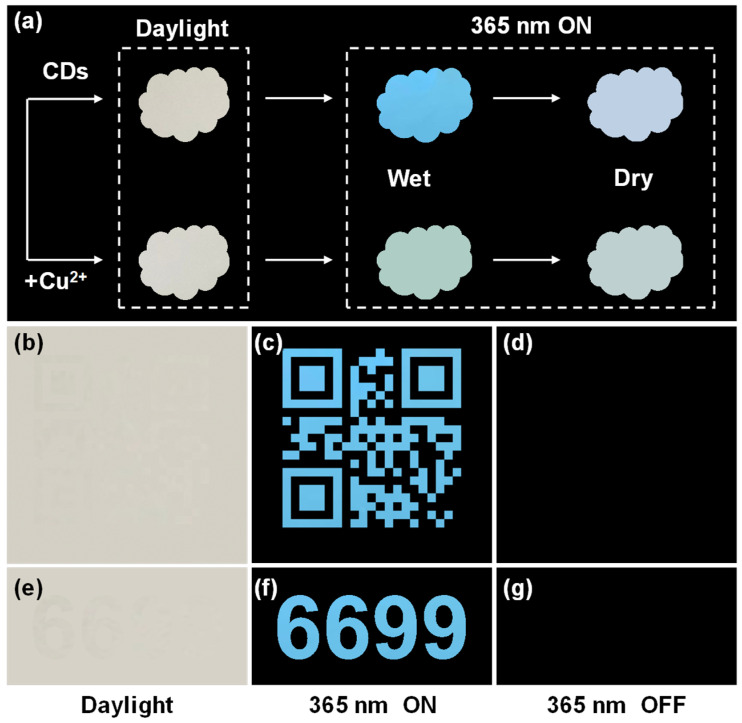
Fluorescent ink application of CDs. (**a**) Illustration of CD dispersion as a security ink in anticounterfeiting applications. Inkjet-printed sample images of (**b**–**d**) QR code and (**e**–**g**) “6699” number in daylight (**b**,**e**), 365 nm UV light ON (**c**,**f**), and OFF (**d**,**g**).

Benefiting from their great photostability and excellent fluorescent properties, synthetic CD dispersion can potentially be employed as an encrypted security ink in anti-counterfeiting and information encryption fields. Fluorescent printing has garnered significant research interest due to its simple operation and effortless verification, distinguishing it from commercial anti-counterfeiting mechanisms such as laser holographic response, temperature response, and magnetic response. Currently, fluorescent inks primarily rely on organic dyes, semiconductor QDs, and rare earth materials for their formulation [51,52]. Nevertheless, the high cost, limited biocompatibility, and potential environmental risks associated with these materials may restrict their utilization. Thus, CDs are regarded as a promising new material for information encryption and anti-counterfeiting. As a proof-of-concept application, samples of various patterns were easily prepared (Figure 7), with a cloud-shaped tag, QR code, and Arabic numbers included. The cloud-shaped tag is pale yellow in daylight and emits bright blue fluorescence under the irradiation of 365 nm UV light (Figure 7a). Even when the tag is dried, it can still emit a slight blue fluorescence. However, once the copper ion solution had been dropped onto a cloud-shaped paper substrate that had been soaked in CD ink, the bright blue fluorescence quickly disappeared and glowed light green. After the paper substrate had dried, the light green color almost disappeared. These phenomena not only indicate that the CDs have a potential application in anti-counterfeiting, but also that they exhibit high sensitivity to copper ions. Similarly, the QR code (Figure 7b) and the “6699” number (Figure 7e) are completely invisible to the naked eyes under natural daylight, while bright blue fluorescent patterns can be observed under the 365 nm UV light (Figure 7c,f) and disappear once the lights is turned off (Figure 7d,g). Thus, it follows that the sample patterns can be designed flexibly, and they can exhibit high covertness under natural daylight. They can emit bright blue fluorescence when subjected to 365 nm UV, even if the sample is sophisticated and imperceptible. These results certify that these CDs have potential uses in high-level anti-counterfeiting fields to identify the authenticity of valuable items (e.g., tickets, merchandise tags, and coupons), as well as in security fields for protecting important information (e.g., sensitive messages, passwords, and documents).

### 2.5. Cytotoxicity and Cell Imaging of CDs

**Figure 8:**
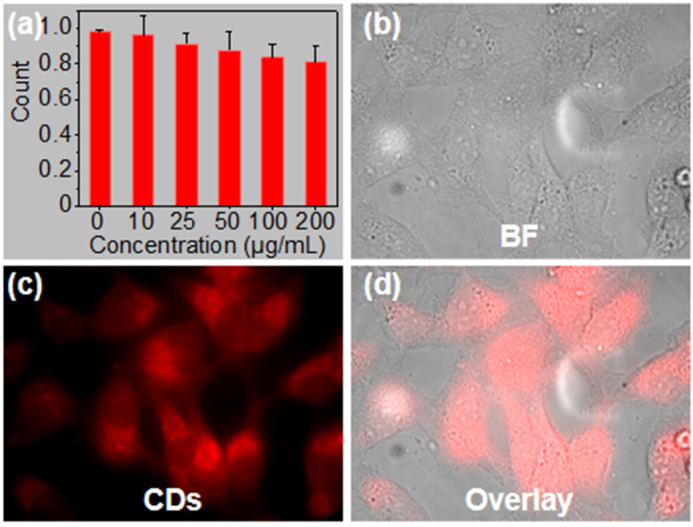
Cytotoxicity and bioimaging of CDs against HeLa cells. (**a**) Cell viability of CDs assessed by using MTT assays. Confocal fluorescence images of HeLa cells incubated with CDs: (**b**) bright field, (**c**) fluorescent channel, (**d**) merging of (**b**,**c**).

To explore the potential of the CDs serving as a probe for bioimaging, the cytotoxicity against HeLa cells was conducted using MTT assays. As described in Figure 8a, the viability of HeLa cells remained almost constant, even when the cells were incubated continuously with a high concentration of 200 μg mL^–1^ CDs for 24 h. The result demonstrates that these CDs have extremely low cytotoxicity and good biocompatibility with HeLa cells. Moreover, the aforementioned emission excited by 532 nm also provides a high-quality guarantee for cell imaging. HeLa cells were incubated with CDs for 5 h and then imaged using a confocal fluorescence microscope under 532 nm laser excitation. The normal morphology and good growth of cells in the bright field also manifested the low cytotoxicity of the CDs. The fluorescence images demonstrate a high uptake of CDs by live cells (Figure 8b) and suggest that CDs can pass through cell membranes and enter cells, mainly becoming located in cytoplasmic regions (Figure 8c,d).

## 3. Materials and Methods

### 3.1. Chemicals

All chemical agents were acquired commercially and utilized without subsequent purification. Diaminomaleonitrile (C_4_H_4_N_4_) and Boc-D-2, 3-diaminopropionic acid (C_8_H_16_N_2_O_4_) were purchased from Macklin (Shanghai, China). Human cervical cancer cells (HeLa) were obtained from the cell bank of the Chinese Academy of Sciences (Shanghai, China).

### 3.2. Synthesis of CDs

First, 0.2 g of Boc-D-2,3-diaminopropionic acid (DPA) and 0.11 g of Diaminomaleonitrile (DAMN) were dissolved in 10 mL of ultrapure water (18.2 MΩ); then, they were transferred to a sealed PTFE reactor (Shanghai Yanzheng Experimental Instrument Co., Ltd., Shanghai, China). The heating oven parameters were programmed by the user. The reaction temperature was set at 200 °C. When the machine was turned on, it reached 200 °C rapidly and maintained that temperature automatically for 5 h. Then, the sample was taken from the reaction vessel when the temperature had naturally cooled to below 80 °C. The product, which was dark brown, was filtered through a 0.22 μm filter to remove the aggregated large particles. Subsequently, the solution was dialyzed with a 500 Da membrane to remove unreacted chemicals and concentrated using a rotary evaporator (Shanghai Kuangsheng Industrial Development Co., Ltd., Shanghai, China). Finally, the samples were lyophilized and diluted for different characterizations and applications.

### 3.3. Material Characterization

Transmission electron microscopic (TEM) images were recorded on a JEM-2100 electron microscope (Jeol., Ltd., Tokyo, Japan) at 200 kV. Ultraviolet–visible (UV–vis) absorption spectra were acquired from 200 to 800 nm using a Shimadzu UV-2450 spectrophotometer (Shimadzu Corporation, Kyoto, Japan). Fluorescence spectra were recorded on the Horiba FluoroMax-4 spectrometer (Horiba Scientific, Kyoto, Japan). Fourier transform infrared (FTIR) spectra were recorded on a NICOLET 6700 IR spectrometer (Thermo Scientific, Waltham, MA, USA). Prior to fluorescence scanning, the spectrophotometer was calibrated using a Xenon lamp scan (Shimadzu Corporation, Kyoto, Japan) and a water Raman scan (Shimadzu Corporation, Kyoto, Japan). After calibration, the xenon lamp peak maximum was at 467 nm, while the water Raman peak maximum occurred at 397 nm for excitation. The fluorescence lifetime was acquired using an FS5 fluorescence spectrometer (Edinburgh instruments, Edinburgh, UK). The emission slit width was set to 0.02. Data acquisition was carried out using the technique of time-correlated single photon counting (TCSPC). All optical measurements were performed at room temperature (~25 °C) under ambient conditions. Single molecule imaging on coverslips or cells was performed on a Nikon total internal reflection fluorescence microscope (TIRFM. Nikon Corporation, Tokyo, Japan).

### 3.4. Luminescence Quantum Yield of CDs

Quantum yields (QYs) of CDs were measured using quinine sulfate in 0.1 M H_2_SO_4_ solution (QY = 58%, 22 °C) as a reference standard. Briefly, the absorbance of the standards and CD samples at the 365 nm excitation wavelength and the corresponding fluorescence spectra of the same solutions were measured, respectively. Integrated fluorescence intensity versus absorbance was calculated and used to determine the quantum yield.

### 3.5. MTT Assays

The human cervical cancer cells, HeLa cells, were cultured in high Dulbecco’s Modified Eagle’s medium (DMEM) supplemented with 10% fetal bovine serum (FBS) in a humidified incubator at 37 °C with the CO_2_ level kept constant at 5%. The cytotoxicity of CDs was evaluated by MTT (3-(4,5)-dimethylthiahiazo (-z-y1)-3, 5-di-phenytetrazoliumromide) assays. Firstly, the cells were seeded in 96-well plates with a density of 5 × 10^3^ cells/well in 100 μL of DMEM containing 10% FBS. The plates were then incubated for 24 h. The cells were washed with PBS buffer, and then 100 μL quantities of fresh medium containing different concentrations of CDs were added. The cells continued to be incubated for 24 h. After that, 20 μL of MTT solution (5 mg/mL) was added to each well, and the plates were further incubated for 4 h at 37 °C. The precipitated formazan was dissolved in 150 μL of dimethyl sulfoxide. The absorbance at 570 nm was measured using a microplate automatic reader (Molecular Devices, M2e. Molecular Devices. San Jose, CA, USA). The percentage cell viability at different concentrations of CDs was illustrated by assigning non-treated cells to 100% viability.

### 3.6. Cell Imaging

Briefly, 200 μL of solution containing approximately 500 HeLa cells was first seeded into an 8-well plate and incubated for 24 h. Before imaging, the cells were washed once with sterile PBS. Then, 200 μL of CDs (100 μg/mL) was added and further incubated for 5 h at 37 °C. Subsequently, the CD-stained cells were rinsed three times with PBS buffer (pH 7.4) and imaged under a Nikon total internal reflection fluorescence microscope (TIRFM). The CDs were excited using a 532 nm laser in conjunction with a 570 nm dichroic mirror and a 593/40 nm band-pass filter. The images were taken with an Andor DU897 EMCCD (16 µm/pixel, Oxford Instruments, Bristol, UK) using 100× objective combined with a 1.5× magnification changer lens, resulting in an effective pixel dimension of approximately 106 nm. The electron-multiplying gain of EMCCD was set to 300 and the pixel readout rate was set to 1 MHz at 16 bits. The excitation density was about 20 W cm^−2^. The exposure time was set to 1 frame. The obtained image series were analyzed with ImageJ (ImageJ1, 1.8.0.345).

### 3.7. Fluorescence Detection of Cu^2+^

Ultrapure water was used to prepare different metal ion solutions, including K^+^, Ca^2+^, Co^2+^, Na^+^, Zn^2+^, Mn^2+^, Ba^2+^, Sr^2+^, Fe^2+^, Cu^2+^, Al^3+^, and Fe^3+^. After adding 100 µL of an equivalent CD solution, the fluorescence spectra of these solutions were recorded under 365 nm excitation to assess the response of ions after incubation for 5 min. Furthermore, to investigate the selectivity and sensitivity of Cu^2+^, various concentrations of Cu^2+^ solution (100, 80, 60, 50, 40, 30, 20, 10, 5, 4, 3, 2, 1, 0.5, and 0.1 μM) were prepared to detect the fluorescence change after the fluorescence quenching of CDs. In addition, the limit of detection (LOD) for Cu^2+^ was calculated based on the following equation:

$$LOD=\frac{3\sigma }{S}$$

where *σ* represents the standard deviation of the analytical blank solution (*n* = 5), and *S* is the slope of the linear fitting equation.

## 4. Conclusions

The present research work successfully reports novel CDs with a high quantum yield of 28.2% produced through an easy hydrothermal method using Diaminom-aleonitrile and Boc-D-2, 3-diaminopropionic acid. The size of the as-prepared CDs is ~7.5 nm and they exhibit chemical functional groups present on their surfaces. Due to their highly water-soluble property, the CDs have been proven to possess selective and sensitive behavior against Cu^2+^ ions because of aggregation-induced emission quenching in CD-Cu^2+^. Meanwhile, it has been demonstrated that CDs have a high quantum yield, excellent photostability, and low cytotoxicity. CDs have been utilized as fluorescence ink in anti-counterfeiting. Moreover, the cell imaging of CDs with low cytotoxicity and good biocompatibility has proved their diffusion ability through HeLa cells. Judging by these results, it can be concluded that these novel CDs could be a promising potential fluorescence probe in the fields of chem-sensors, bio-sensors, and anti-counterfeiting.

## Figures and Tables

**Table 1:** Detection of Cu^2+^ using different CDs in previous reports.

Chemo-Sensors	Starting Materials	QYs (%)	Fluorescence	Linear Range (mM)	LOD (μM)	Ref.
G-CDs	p-phenylenediamine, thiosemicarbazone	28.26	Greenish	0.02–0.2	4.8	[31]
N, S, O-CDs	L-cysteine		Blue	0.01–0.0333	2.0	[47]
CDs-SH	Water hyacinth	13.00	Blue	0–0.6	31.1	[40]
NS-CDs	L-cysteine	18.00		0–0.5	5.0	[48]
NH_2_-CQDs	Lignin	21.10	Greenish	0.0005–0.08	2.420	[49]
N-CDs	p-phenylenediamine, polyethylene glycol 20000	2.16	Red	0.045–0.07	45.87	[49]
P, Br-CDs	Phenylenediamine, disodium hydrogen phosphate, KBr	11.30	Red	0–0.15	4.408	[50]
CDs	Diaminomaleonitrile, Boc-D-2, 3-diaminopropionic acid	28.2	Blue	0.0001–0.009	1.34	This work

## Data Availability

The data presented in this study are available wholly within the manuscript.
